# Iguratimod as a New Drug for Rheumatoid Arthritis: Current Landscape

**DOI:** 10.3389/fphar.2020.00073

**Published:** 2020-02-26

**Authors:** Sisi Xie, Shu Li, Jing Tian, Fen Li

**Affiliations:** Department of Internal Medicine, The 2nd Xiangya Hospital of Central South University, Changsha, China

**Keywords:** iguratimod, rheumatoid arthritis, NF-kappa B, randomized controlled trial, pharmacology

## Abstract

Iguratimod (IGU) is a novel synthetic small molecule disease modified anti-rheumatic drug approved only in Japan and China up to date. IGU plays an important immunomodulatory role in the synovial tissue of rheumatoid arthritis by inhibiting the production of immunoglobulins and cytokines and regulating T lymphocyte subsets. IGU also regulates bone metabolism by stimulating bone formation while inhibiting osteoclast differentiation, migration, and bone resorption. In clinical trials, IGU was shown to be superior to placebo and not inferior to salazosulfapyridine. Combined therapy of IGU with other disease-modifying anti-rheumatic drugs showed significant improvements for disease activity. IGU has good efficacy and tolerance as an additional treatment for rheumatoid arthritis patients with inadequate response to methotrexate and biological disease-modifying anti-rheumatic drugs. In this review, we summarize current landscape on the mechanism of action of IGU and its clinical effectiveness and safety. It is expected that further translational studies on IGU will pave the road for wider application of IGU in the treatment of autoimmune diseases other than rheumatoid arthritis.

## Introduction

Rheumatoid arthritis (RA) is a chronic autoimmune disease of unknown etiology, and it is characterized by joint synovitis, progressive bone destruction, and loss of joint function. The occurrence of RA is related to genetic, environmental, and immune factors. The goal of the treatment for patients with RA is clinical remission or low disease activity, ultimately preventing joint function damage. Current treatments for RA include non-steroidal anti-inflammatory drugs (NSAIDs), conventional synthetic disease-modifying anti-rheumatic drugs (csDMARDs), and biological disease-modifying anti-rheumatic drugs (bDMARDS). These drugs can reduce the activity of the disease, but have some shortcomings such as serious complications of infection or high cost of treatment.

Iguratimod (IGU or T-614) is a novel synthetic small molecule disease modified anti-rheumatic drug approved only in Japan and China. IGU can inhibit nuclear factor-kappa B (NF-κB) activation by interfering with NF-κB translocation from the cytoplasm to the nucleus without affecting the degradation of Ikappa Balpa in lipopolysaccharide-stimulated THP-1 cells (human monocytic leukemia cell line) (Aikawa et al., 2002). Similar results were also confirmed in cultured human synovial cells and the rat alveolar macrophage (Kohno et al., 2001; Jiang et al., 2006). Other studies in macrophages and microglia showed that IGU inhibited nuclear translocation of NF-κB 65 and pro-inflammatory response (Li et al., 2018). Based on the above evidences, IGU is widely used as a new csDMARD in China and Japan for RA treatment possibly by directly inhibiting the production of immunoglobulins and a variety of inflammatory cytokines (Tanaka et al., 2003). In addition, IGU can promote bone formation and inhibit bone resorption (Gan et al., 2016; Wang et al., 2017). A number of clinical studies have reported the efficacy and safety of IGU monotherapy, combination therapy, and as an add-on therapy (**Tables 1**, **2**). Therefore, in this review, we summarize recent advances in our understanding of the mechanism of action of IGU and its clinical effectiveness and safety.

**Table 1:** Characteristics of the clinical trials with iguratimod for RA.

References	Study	Participants	Number	Intervention	Duration	Primary Outcomes
Hara et al., 2007	Randomized controlled double-blind experiment	Country: Japan Site: Multicenter	Total: 376 IGU group: 147 SASP group: 156 PlA group: 73	IGU group: 25 mg QD (0–4 weeks) 25 mg BID (4–24 weeks) SASP group: 500 mg BID (0–28 weeks) Placebo group: placebo (0–28 weeks)	28 weeks	ACR 20 at 24 Weeks: IGU was superior to placebo (P < 0.0001), not inferior to SASP.
Lü et al., 2008	Randomized controlled double-blind experiment	Country: China Site: Multicenter	Total: 280 IGU group 1: 92 IGU group 2: 93 PlA group: 95	IGU group 1: 25 mg QD (0–24 weeks) IGU group 2: 25 mg BID (0–24 weeks) PlA group: placebo (0–24 weeks)	24 weeks	ACR 20 and ACR 50 at 24 Weeks: IGU treatment group was significantly better than that in the placebo group (P < 0.0001).
Lu et al., 2009	Randomized controlled double-blind experiment	Country: China Site: Multicenter	Total: 489 IGU group 1: 163 IGU group 2: 163 MTX group: 163	IGU group 1: 25 mg QD (0–4 weeks) 25 mg BID (4–24 weeks) IGU group 2: 25 mg BID (0–24 weeks) MTX group: 10 mg QW (0–4 weeks) 15 mg QW (4–24 weeks)	24 weeks	ACR 20 at 24 Weeks: IGU group 2 (63.8%) was not statistically significantly different from that for patients receiving MTX treatment (62.0%), and was superior to that for patients in IGU group 1 (50.9%). The result of the noninferiority analysis indicated that the efficacy of IGU group 2 was not lower than that of MTX by <10%.
Ishikawa and Ishikawa, 2019	Retrospective study	Country: Japan Site: Single-center	Total: 213	IGU 25 mg QD (0–4 weeks), then increased to 25 mg BID based on the physician’s discretion.	104 weeks	DAS and SDAI disease activity States showed statistically significant improvement (P < 0.001).
Nozaki et al., 2019	Retrospective study	Country: Japan Site: Single-center	Total: 197	The patients took IGU at a dose of 25–50 mg or SASP at a dose of 500–1,000 mg once daily or 2×/day, in the morning and/or evening	3 years	At month 36, the retention rates of the IGU and SASP groups were 52.4% vs. 32.1%. The rate of responders (good or moderate response) at month 36 was 85.8% vs. 65.2% in the IGU and SASP groups, respectively.

**Table 2:** Characteristics of the clinical trials of iguratimod in combination for RA.

Hara et al., 2014	Randomized double-blind placebo-controlled trial	Country: Japan Site: Multicenter	Total: 252 IGU+MTX group: 165 (PLA/IGU)+MTX group: (Weeks 1–28): 88 (Weeks 28–52): 68	IGU+MTX group: IGU 25 mg BID, MTX 6 or 8 mg QW, and folic acid 5 mg QW (0–52 weeks). (PLA/IGU)+MTX group: Pla tablets (1–28 weeks); MTX 6 or 8 mg QW, and folic acid 5 mg QW (1–52 weeks); IGU 25 mg QD (28–32 weeks), 25 mg BID (32–52 weeks).	52 weeks	ACR20 at week 52: IGU+MTX group was similar to that at week 24 (69.5%). (PLA/IGU)+MTX group, the switch to IGU treatment significantly improved from 30.7% at week 24 to 72.1% at week 52. ACR50, ACR70 at week 52: IGU+MTX group was significantly improved compared with the values at week 24.
Ishiguro et al., 2013	Randomized double-blind placebo-controlled trial	Country: Japan Site: Multicenter	Total: 252 IGU group: 164 Placebo group: 88	IGU group: 164 IGU 25 mg QD (0–4 weeks) 25 mg BID (4–24 weeks). MTX 6 or 8 mg QW, folic acid 5 mg QW. Placebo group: MTX 6 or 8 mg QW and folic acid 5 mg QW,and placebo tablets	24 weeks	ACR20 at week 24 was 69.5% in the IGU group compared with 30.7% in the placebo group (P < 0.001). Significant improvements in the ACR50, ACR70.
Xia et al., 2016	Prospective trial	Country: Japan Site: Single-center	Total: 131 MTX+IGU group: 44 IGU group: 38 MTX group: 49	IGU group: 25 mg BID (0–24 weeks) MTX group: 10 mg QW (0–24 weeks) MTX+IGU group: IGU 25 mg BID (0–24 weeks). MTX 10 mg QW (0–24 weeks)	24 weeks	ACR 20 and ACR 50 at 24 weeks: combination of IGU with MTX was superior to IGU or MTX monotherapy.
Duan et al., 2015	Randomized controlled trial	Country: China Site: Single-center	Total: 60 MTX+ IGU group: 30 MTX group: 30	MTX+ IGU group: IGU 25 mg BID (0–24 weeks). MTX 10 mg QW (0–4 weeks), 12.5 mg QW (4–24 weeks) MTX group: 10 mg QW (0–4 weeks), 12.5 mg QW (4–24 weeks)	24 weeks	ACR50 at 24 weeks: MTX+T-614 group showed statistically significant differences comparing with the MTX group (P < 0.05).
Yoshikawa et al., 2018	Retrospective study	Country: Japan Site: Single-center	Total: 41 patients who showed an inadequate response to biological DMARDs	IGU 25 mg QD (0–4 weeks), then increased to 25 mg BID based on the physician’s discretion.	24 weeks	Remission can be achieved by IGU addon in RA patients responding partially to 24-week or longer administration of bDMARD.
Zheng et al., 2018	Retrospective study	Country: China Site: Single-center	Total: 23 patients who showed an inadequate response to MTX–CsA–HCQ– prednisone	MTX: 12.5 mg QW HCQ: 0.1 mg BID CsA: 50 mg BID Prednisone: 7.5 mg QD IGU: 25 mg BID	24 weeks	After 24 weeks the RA patients showed a significant improvement in mean DAS28 score from baseline. 18 (78%), 15 (65%), and 12 (50%) patients, respectively, met the ACR20, ACR50, and ACR70 response criteria.
Ebina et al., 2019	Retrospective study	Country: Japan Site: Multicenter	Total: 31 patients who showed an inadequate response to TCZ	TCZ 162 mg Q2W or 8 mg/kg QM; IGU 25 mg QD, then increased to 25 mg BID depending on physician’s decision.	24 weeks	Using the EULAR criteria, 64.5% achieved a moderate response, and 51.6% achieved ACR20 at 24 weeks.
Suto et al., 2019	Retrospective study	Country: Japan Site: Multicenter	Total: 69 IGU group: 28 MTX+IGU: 28 bDMARDs+IGU: 13	IGU group: IGU 25 mg QD (0–4 weeks), then increased to 25 mg BID based on the physician’s discretion. MTX+IGU group: MTX was 8.5 ± 3.4 mg/week bDMARDs+IGU group: IFX/ETN/ADA/TCZ/ABT/GLM(n=1/4/3/1/2/2)	36 months	The survival rate of IGU therapy at 3 years was 40.6%. The disease activity was significantly decreased in the IGU group and MTX plus IGU group compared with the baseline.

## The Mechanism of Action of IGU

### Anti-Inflammation and Analgesia

IGU was originally developed as a novel NSAIDs. In 1992, Tanaka et al. reported the anti-inflammatory, analgesic, and antipyretic effects of IGU in different animal models (Tanaka et al., 1992c), and the mechanism was related to the inhibition of the metabolism of arachidonic acid metabolite prostaglandin E2 (Tanaka et al., 1992b), the inhibition of the release of bradykinin (Tanaka et al., 1992d), the production of interleukin(IL)-1 and IL-6 (Tanaka et al., 1992a), and selective inhibition of the activity of cyclooxygenase-2 (Tanaka et al., 1995). In some autoimmune disease models, IGU exhibited significant inhibitory effects in experimental autoimmune encephalitis, chronic contractile injury with neuropathic pain, and dextran sulphate sodium-induced colitis (Morimoto et al., 2017; Jiang et al., 2018; Li et al., 2018).

In synovial cells, IGU can significantly inhibit the expression of cytokines including IL-6, IL-8, granulocyte colony-stimulating factor, and granulocyte macrophage colony-stimulating factor induced by interferon-γ, IL-1β, or 12-O-tetradecanoyl phorbol 13-acetate, and IGU can alleviate the expression of costimulatory molecules including CD54, CD58, CD106, Human Leukocyte Antigen-DR, IGU also significantly inhibited synovial cell-mediated antigen-specific T cell proliferation (Kawakami et al., 1999). In addition, IGU inhibited the upregulation of IL-6, IL-8, and monocyte chemoattractant protein 1 induced by tumor necrosis factor alpha (TNF-α) in RA synovial cells in a concentration dependent manner (Kohno et al., 2001).

### Regulation of Immune Response

CD4 + T cells and activated B lymphocytes play an important role in the chronic inflammation of RA. IGU can exert immunomodulatory effects on these cells during the progression of RA.

#### Regulating T Lymphocyte Subsets

The pathogenesis of RA involves chronic inflammatory response of autoreactive T cells. T cell lineages include Th1, Th17 cells, CD4 + CD25 + regulatory T cells (Treg) and follicular helper T cells (Tfh). A clinical study showed that Th1 and Th17 were downregulated while Treg was upregulated in patients with RA after IGU treatment, accompanied by decreased levels of Th1, Th17, Tfh associated inflammatory cytokines and transcription factors, and increased levels of Treg associated cytokines and transcription factors (Xu et al., 2015). In a mouse model of dextran sulphate sodium-induced-induced colitis, IGU relieved the symptoms of colitis and reduced intestinal tissue damage, perhaps due to the downregulation of Th17 cells and the upregulation of Treg cells (Jiang et al., 2018).

In addition, IGU has a significant protective effect on cartilage and bone erosion in a rat model of collagen-induced arthritis by distorting Th17-driven response and inhibiting the production of anti-type II collagen antibodies (Du et al., 2008). IGU can inhibit IL-17 signal pathway by reducing the mRNA stability and MAPK phosphorylation, targeting Act1, and disrupting the interaction of Act 1 with TRAF5 and Ikki (Luo et al., 2013).

#### Regulating Humoral Immunity

In 2003, Tanaka et al. first discovered that IGU directly inhibited B lymphocytes in mouse and human to reduce the production of immunoglobulins although it had no effect on the proliferation or apoptosis of B cells (Tanaka et al., 2003). IGU could reduce circulating plasma cells with a non-anti-proliferative mechanism in MRL/LPR mice (Yan et al., 2014). Recently, Ye et al. demonstrated that IGU did not affect the activation and proliferation of B cells in the established *in vitro* human antibody secreting cell differentiation system, but inhibited the differentiation of human antibody-secreting cell by targeting the protein kinase C (PKC) and early growth response 1 (EGR1) axis (Ye et al., 2019).

### Regulation of the Metabolism of Bone and Cartilage

#### Promoting Bone Formation

Kuriyama et al. first found that IGU could promote osteoblastic differentiation of stromal cell line and preosteoblastic cell line *in vitro* and bone formation induced by recombinant human bone morphogenetic protein-2 *in vivo* (Kuriyama et al., 2002). Another study demonstrated that IGU promoted osteoblast differentiation by upregulating DLX5 and the phosphorylation of p38, increasing Osterix expression, and inhibiting phosphorylated NF-κB levels (Song et al., 2018). Moreover, clinical studies found that IGU and methotrexate (MTX) combined therapy was more effective in stimulating bone formation (Ishiguro et al., 2013).

#### Inhibiting Bone Absorption

It was first discovered in the rat with collagen-induced arthritis model that IGU could inhibit cartilage and bone erosion and protect joint integrity (Du et al., 2008). Subsequently, *in vitro* studies revealed that IGU significantly inhibited nuclear factor κB ligand (RANKL)-induced RAW264.7 cell differentiation and migration and bone resorption. The mechanism was related to inhibiting the activation of osteoclastogenesis through MAPKs and NF-κB pathways (Gan et al., 2016). Clinical studies showed that IGU would counteract bone resorption by regulating the RANKL/RANK/osteoprotegerin (OPG) system without influencing Dickkopf-1 levels (Wang et al., 2017). In IL-6-stimulating rheumatoid arthritis synovial fibroblasts, IGU reduced RANKL expression and RANKL/OPG ratio, and ERK 1/2 pathway may be involved in the regulation of RANKL expression by IGU (Wei et al., 2015).

IGU also played protective role in the model of benign bone loss induced by oophorectomy. The mechanism may be through the inhibition of peroxisome proliferator-activated receptor-γ/c-fos in RANKL-induced osteoclastogenesis (Wu et al., 2017). In addition, IGU can reduce bone damage caused by tumors, but has little effect on tumor cell proliferation and invasion (Sun et al., 2016).

#### Preventing Cartilage Erosion

Matrix metalloproteinases (MMPs) are produced by fibroblast-like synoviocytes and play an important role in the destruction and erosion of articular cartilage in RA. IGU inhibits the production of MMP-1 and MMP-3 by rheumatoid synovial fibroblasts, thereby inhibiting the invasion of fibroblast-like synoviocytes stimulated by inflammatory cytokine (Du et al., 2012). Clinical study reported that serum MMP-3 level predicted the response in RA patients with IGU and bDMARDs combined treatment (Tokai et al., 2018).

### Summary

**Figure 1:**
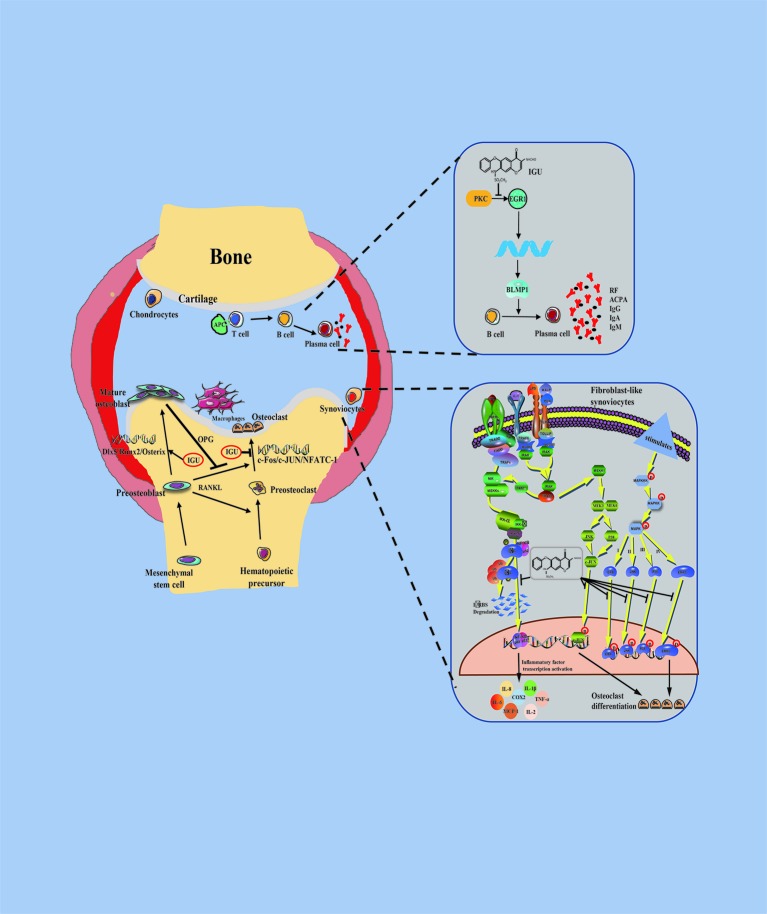
Schematic representation of pharmacological actions of iguratimod.
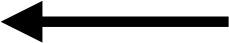
 Stimulation by iguratimod 
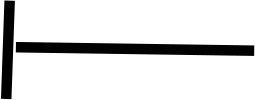
 Inhibition by iguratimod.

Taken together, IGU can significantly inhibit the initiation and progression of RA by multiple mechanism such as regulating T cell differentiation, reducing the production of pro-inflammatory cytokines and immunoglobulins, promoting bone formation, and inhibiting bone resorption (**Figure 1**).

## Clinical Application of IGU

### Materials and Methods

Clinical studies published up to May 2019 were searched *via* database (PubMed). The key words are iguratimod and rheumatoid arthritis. The types of studies included in the trials included all randomized controlled trials, non-randomized controlled trials, and retrospective studies published in all journals. Participants were patients with RA who met the clinical diagnostic criteria of the American Rheumatism Association, including patients with refractory rheumatoid arthritis. Intervention types included comparisons of IGU therapy (monotherapy or combination with other DMARDs) versus placebo or other DMARDs, with a study duration of at least 24 weeks. Results must include at least one of the following: American College of Rheumatology (ACR) response rate of 20, 50, or 70% or disease activity score for 28 joint counts based on red blood cell sedimentation rate (DAS28-ESR). For adverse events and safety, the study must have the following data: adverse events, serious adverse events, drug discontinuation, and mortality. Sample size was not considered an inclusion or exclusion criterion. Studies published in language ​​other than English were excluded. The initial search retrieved 44 articles from PubMed. After applying the filter, a total of 16 articles remained.

### RA Treatment

#### Phase I Clinical Study

The results of Phase I clinical trials suggested that in healthy adult volunteers IGU was well tolerated in the 3.125–50 mg dose range and caused no serious adverse reactions. There was no significant difference in T_max_, T_1/2_, K_a_, and V/F between IGU at 25, 50, and 75 mg. AUC_0-last_ and C_max_ were linear in the range of 25–75 mg. There was no accumulation in repeated administration. In addition, food intake had no effect on the bioavailability of IGU, and could promote the absorption of IGU (Xiao F. et al., 2018).

#### Phase II Clinical Study

In a 24-week, double-blind, randomized, placebo-controlled, and multi-center clinical study, American College of Rheumatology (ACR) 20, ACR50, and ACR70 were significantly more effective in IGU treatment groups than in placebo group within 8 weeks of treatment. At the end of 24 weeks of treatment, ACR 20 among IGU groups of 25 mg/d and 50 mg/d and placebo group were 39.13, 61.29, and 24.21%, respectively, and ACR50 among three groups were 23.91, 31.18, and 7.37%, respectively. There were also significant differences in erythrocyte sedimentation rate (ESR) and C-reactive protein (CRP) between the two dosages of treatment group, and the incidence of adverse reactions was a little higher in IGU groups than in placebo group (**Table 1**) (Lü et al., 2008).

#### Phase III Clinical Study

Another 28-week, multicenter, randomized, double-blind study showed that IGU at dosage of 50 mg/d was superior to placebo in ACR20 (53.8% vs. 17.2%; P < 0.001), not inferior to salazosulfapyridine (SASP) (63.1% vs. 57.7%, 95% confidence interval was −7.9%~18.7%). There was no significant difference in the incidence of adverse events and adverse reactions between IGU and SASP (P > 0.05) (**Table 1**) (Hara et al., 2007). Another randomized controlled trial showed that IGU at dosage of 50 mg/d was effective and well tolerated in RA patients (**Table 1**) (Lu et al., 2009).

#### Post-Marketing Clinical Study

Mimori et al. conducted a 52-week post-marketing surveillance study in Japanese and mid-term safety analysis of 2,679 patients at week 24. The total incidences of AES, ADRs, and severe ADRs were 38.41, 31.65, and 3.21%, respectively. The most common adverse reactions are liver dysfunction and gastrointestinal disease. Most common liver dysfunction events are abnormal laboratory test values. Patients tend to recover during or after IGU administration. The most common serious adverse reactions include bacterial pneumonia, interstitial lung disease, and pneumocystis pneumonia. In patients administrated with warfarin and IGU in combination, IGU interacted with warfarin to produce serious adverse events, including alveolar hemorrhage and elevated internationally standardized prothrombin time ratio (PT-INR), suggesting that IGU can improve anticoagulant activity of warfarin (Mimori et al., 2017). However, the incidence of adverse reactions peaked around 4 weeks of treatment. Over the next 28 weeks, the incidence of adverse reactions did not increase but decreased. Regarding the combined use of IGU and warfarin, no clinically important findings have been identified since the interim report. Based on a significant reduction in DAS28-CRP from baseline to 52 weeks, an improvement in RA disease activity was observed, confirming the efficacy of IGU (Mimori et al., 2019). Another 104-week retrospective analysis found early and sustained efficacy in group of IGU, and the use of NSAIDs and oral prednisolone had higher incidence of gastric bleeding or gastric perforation than that of IGU (**Table 1**) (Ishikawa and Ishikawa, 2019). A 3-year study showed that the 3-year survival rate for IGU alone was 40.6%. The disease activity of IGU combined with MTX at the end of 3 years was significantly lower than that at the baseline, and 38 RA patients (55.1%) with combined therapy developed low disease activity at last (**Table 2**) (Suto et al., 2019).

In addition, a recent genetic prediction study found that the *ABCG2* rs2231142 A allele responded better to IGU, but the NAT2 rs1495742G vector had a lower response to IGU. CYP2C19*2 rs4244285 A carriers had a higher risk of IGU toxicity than GG carriers (Xiao W. et al., 2018). Further genetic polymorphism studies help predict the efficacy and toxicity of IGU in RA patients.

#### IGU Combined With Other Drugs

##### IGU Combined With MTX

Two 24-week clinical studies both showed similar results. The group of IGU combined with MTX had better outcomes than that of MTX alone, such as Patient's clinical manifestations including joint tenderness and swollen joint count, pain visual analogue scale overall assessment of patients and physicians, ESR, CRP, health assessment questionnaire, DAS28, simplified disease activity index, and ACR 50 (P < 0.05) (**Table 2**) (Duan et al., 2015; Xia et al., 2016). Interestingly, there was no significant difference in the incidence of adverse events between the IGU+MTX group and the MTX group (P > 0.05). In the study of patients with active RA who had poor efficacy with MTX alone, the response of IGU plus MTX group was significantly better than that of placebo plus MTX group at the end of 24 weeks (P < 0.001) (**Table 2**) (Ishiguro et al., 2013). Another study showed that the efficacy and tolerability of IGU in combination with MTX maintained for more than 52 weeks in patients with active RA who did not respond adequately to MTX (**Table 2**) (Hara et al., 2014).

##### IGU Combined With csDMARDs

A retrospective study reported that patients with refractory RA after 24 weeks of MTX- Cyclosporin A-Hydroxychloroquine-Prednisone combined therapy were administered with stable dose of IGU (25 mg twice a day). There were significant differences in DAS28, ESR, CRP, and health assessment questionnaire between the end of 24 weeks and baseline (P < 0.05), and no serious adverse events were reported within 24 weeks (**Table 2**) (Zheng et al., 2018).

##### IGU Combined With bDMARDs

In a retrospective study, IGU was used in RA patients inadequately responding to 24-week or longer with bDMARDs, and DAS28-ESR decreased significantly from baseline to 24 weeks (p < 0.001). Overall, 38.3% RA patients achieved clinical remission, and ultrasound evaluations were performed with similar results (p < 0.001) (**Table 2**) (Yoshikawa et al., 2018). In another multicenter study of RA patients with insufficient response to tocilizumab and other csDMARDs (MTX, SASP, and tacrolimus), the addition of IGU may be an effective complementary treatment, because outcome measures including DAS28-CRP and clinical disease activity index had been improved significantly and 51.6% RA patients achieved ACR 20 at 24 weeks (**Table 2**) (Ebina et al., 2019). These two studies indicated that IGU add-on therapy can be a therapeutic strategy to achieve remission in RA patients inadequately responding to 24-week treatment with bDMARDs.

### Treatment of Other Rheumatic Diseases

IGU can significantly reduce the production of IL-17 and TNF-α, which play an important role in the pathogenesis of Axial Spondyloarthritis (axSpA) inflammation. Some retrospective case studies have shown that IGU has good effects on disease activity and inflammatory markers in patients with refractory axSpA (Luo et al., 2018). In addition, IGU improved immune nephritis in MRL/LPR mice by a non-anti-proliferative mechanism, suggesting that IGU has a potential therapeutic effect on systemic lupus erythematosus (Yan et al., 2014).

## Discussion

Pharmacological effects of IGU have been reviewed, including anti-inflammatory and analgesic effects, immunoregulation, and bone metabolism, which may be mediated by inhibiting the activation of NF-κB (Tanaka et al., 2015). In this review, we summarize recent findings on pharmacological effects of IGU. 1. For anti-inflammatory analgesia, the role of IGU is not limited to various arthritis models, animal models of autoimmune encephalomyelitis, and chronic contraction. IGU also has good effects on neuropathic pain in rats with injury and dextran sulphate sodium-induced colitis in mice. 2. For immunoregulation, IGU can inhibit the production of globulin by B lymphocytes. In clinical studies, the concentrations of immunoglobulins (such as IgG, IgM, and IgA and RF) in the blood showed significant decreases after IGU treatment. IGU does not affect the activation and proliferation of B cells, but directly inhibits B lymphocytes to produce immunoglobulins by targeting the PKC/Egr1/BLIMP1 axis to inhibit human antibody-secreting cell differentiation. In addition, IGU regulates T lymphocyte subsets. A clinical study showed that Th1 and Th17 were downregulated while Treg was upregulated in RA patients treated with IGU, and Treg associated cytokines and transcription factors were upregulated. 3. For bone metabolism, in addition to promoting bone formation, IGU also inhibits bone resorption and prevents cartilage erosion. These pharmacological effects of IGU have been confirmed in clinical trials. Bone erosion can be evaluated by X-rays, and most patients have slow disease progression after treatment. However, future studies with larger sample size and longer observation time are necessary to evaluate the inhibitory effect of IGU on bone erosion. In addition to bone protection in RA, IGU also plays a role in benign bone loss models and tumor-induced bone destruction. In a recent review by Li et al., IGU not only reduces inflammation associated with biomaterials, but also reduces its rejection rate. Therefore, IGU can be used to repair coatings on the surfaces of biomaterials, such as orthopedic implants, orthodontics, maxillofacial surgery, and cardiovascular stents (Li et al., 2019). 4. In addition, Lin et al. recently reported that the therapeutic effect of IGU on RA may be partly due to the regulation of invasive behavior and apoptosis of rheumatoid arthritis-fibroblast-like synoviocytes (RA-FLSs): on one hand, IGU inhibits JNK and P38 MAPK signaling pathways and downregulates the expression of downstream phosphorylated activating transcription factor 2, thereby reducing the production of multiple inflammatory cytokines and matrix metalloproteinases, and inhibiting the proliferation, migration, and invasion of RA-FLSs; on the other hand, IGU activates caspase dependent pathway to promote the apoptosis of RA-FLSs (Lin et al., 2019). Therefore, IGU may reduce arthritis and delay bone damage by inhibiting the invasion and promoting the apoptosis of FLSs.

Furthermore, we have summarized all clinical studies of IGU published in English literatures. In the phase I-IV clinical trials of RA patients, IGU has been proven to be better than placebo but not inferior to sulfadiazine. In the study of the efficacy of IGU and SASP as first-line drugs for RA, at 36 months, the retention rates of IGU and SASP groups were 52.4 and 32.1%, and the effective rates were 85.8 and 65.2%, respectively. PSL use rates were 16.7 and 46.7%, PSL doses were 0.3 mg/d and 2.0 mg/d, and cumulative adverse event rates were 19.8 and 29.2%, respectively. These data indicate that IGU is an effective first-line csDMARDs treatment for RA patients (**Table 1**) (Nozaki et al., 2019). IGU combined with MTX showed better efficacy than IGU or MTX monotherapy. In addition, IGU has good efficacy and tolerability as an additional treatment for RA patients with insufficient response to MTX and biologics. Based on these results, the long-term safety and effectiveness of IGU in patients with RA have been confirmed in clinical studies in Japan. IGU has become a new option for the treatment of RA, and is listed as a DMARDs drug recommended by the Asia Pacific Alliance for Rheumatology Association (APLAR) in the treatment guidelines.

However, current studies on IGU have certain limitations. 1. The current clinical data sources are mainly China and Japan. The study population is mainly East Asians, without other ethnic groups. 2. The direct targets of IGU are not very clear. Current studies on the mechanisms of anti-inflammation, immunity regulation, and bone metabolism are limited, and the actual targets are still unknown. 3. The clinical studies on this drug are mainly short-term, there are no long-term clinical data for more than 3 years. Therefore, multi-center and long-term safety data and comparisons of the safety and effectiveness of IGU with other drugs are necessary. In addition to RA, the anti-inflammatory and anti-rheumatic effects of IGU are shown in other autoimmune diseases, such as Sjogren's syndrome, ankylosing spondylitis, systemic lupus erythematosus, multiple sclerosis, and IgG4-related diseases, which are mainly reported in China. Therefore, more data on the efficacy and safety of IGU in the treatment of other autoimmune diseases should be reported, especially those related to high immunoglobulin. Moreover, since IGU can reduce the inflammation associated with biomaterials and the rejection rate, we hope that more data on the use of IGU for biomaterials will be reported.

In summary, IGU is a new synthetic disease-modifying anti-rheumatic drug. We hope that IGU will not only be used in China and Japan, but also become an effective choice for patients with RA worldwide.

## Author Contributions

FL, SX, and SL contributed conception and design of the study. FL organized the database. SX performed the statistical analysis. SX wrote the first draft of the manuscript. FL, SX, SL, and JT wrote sections of the manuscript. All authors contributed to manuscript revision, read and approved the submitted version.

## Funding

This study was supported by the Natural Science Foundation of Hunan Province of China (2019JJ50895), the Simcere Clinical Research Rheumatoid project of China International Medical Exchange Foundation (Grant Z-2014-06-2-1619), Hunan Provincial Health Committee 225 Talent Project.

## Conflict of Interest

The authors declare that the research was conducted in the absence of any commercial or financial relationships that could be construed as a potential conflict of interest.

## Abbreviations

ACPA, Anticitrullinated antibodies; APC, Antigen presenting cell; COX-2, Cyclooxygenase-2; EGR1, Early growth response 1; IGU, Iguratimod; IL: Interleukin; MCP-1, Monocyte chemoattractant protein-1; NF-κB, Nuclear factor kappa-B; OPG, Osteoprotegerin; P, Phosphorylation; PKC, Protein kinase C; RA, Rheumatoid arthritis; RANKL, Receptor activator of the NF-κB ligand; RF, Rheumatoid factors; TNF, Tumor necrosis factor.
