# Corrigendum: Iguratimod as a New Drug for Rheumatoid Arthritis: Current Landscape

**DOI:** 10.3389/fphar.2020.00488

**Published:** 2020-04-08

**Authors:** Sisi Xie, Shu Li, Jing Tian, Fen Li

**Affiliations:** Department of Internal Medicine, The 2nd Xiangya Hospital of Central South University, Changsha, China

**Keywords:** iguratimod, rheumatoid arthritis, NF-kappa B, randomized controlled trial, pharmacology

In the original article, there was a mistake in **Table 2** as published. The row headers of **Table 2** in the article are missing. The corrected **Table 2** appears below.

**Table 2:** Characteristics of the clinical trials of iguratimod in combination for RA.

References	Study	Participants	Number	Intervention	Duration	Primary Outcomes
Hara et al., 2014	Randomized double-blind placebo-controlled trial	Country : Japan Site: multicenter	Total: 252 IGU+MTX group:165 (PLA/IGU)+MTX group: (Weeks 1-28):88 (Weeks28–52):68	IGU+MTX group: IGU 25mg Bid,MTX 6 or 8 mg Qw and folic acid 5mg Qw(0-52 weeks). (PLA/IGU)+MTX group: Pla tablets(1-28 weeks); MTX 6 or 8mg Qw and folic acid 5 mg QW(1-52 weeks);IGU 25mg Qd(28-32 weeks),25mg Bid (32-52 weeks).	52 weeks	ACR20 at week 52:IGU+MTX group was similar to that at week 24 (69.5%).(PLA/IGU)+MTX group, the switch to IGU treatment signficantly improved from 30.7% at week 24 to 72.1% at week 52. ACR50, ACR70 at week 52: IGU+MTX group was significantly improved compared with the values at week 24.
Ishiguro et al., 2013	Randomized double-blind placebo-controlled trial	Country : Japan Site:multicenter	Total: 252 IGU group:164 placebo group:88	IGU group:164 IGU 25mg Qd (0-4 weeks) 25mg Bid (4-24 weeks). MTX 6 or 8mg Qw, folic acid 5mg Qw. placebo group: MTX 6 or 8mg Qw and folic acid 5mg Qw,and placebo tablets	24 weeks	ACR20 at week 24 was 69.5% in the IGU group compared with 30.7% in the placebo group (P < 0.001). Significant improvements in the ACR50, ACR70.
Xia et al., 2016	Prospective trial	Country : Japan Site: Single-center	Total: 131 MTX+IGU group:44 IGU group:38 MTX group:49	IGU group:25mg Bid(0-24 weeks) MTX group:10mg Qw (0-24 weeks) MTX+IGU group: IGU 25mg Bid (0-24 weeks).MTX 10mg Qw (0-24 weeks)	24 weeks	ACR 20 and ACR 50 at 24 weeks: combination of IGU with MTX was superior to IGU or MTX monotherapy.
Duan et al., 2015	Randomized controlled trial	Country : China Site : Single-center	Total: 60 MTX+ IGU group:30 MTX group:30	MTX+ IGU group: IGU 25mg Bid (0-24 weeks).MTX 10mg Qw (0-4 weeks),12.5mg Qw (4-24 weeks) MTX group: 10mg Qw (0-4 weeks),12.5mg Qw (4-24 weeks)	24 weeks	ACR50 at 24 weeks:MTX+T-614 group showed statistically significant differences comparing with the MTX group (P < 0.05).
Yoshikawa et al., 2018	Retrospective study	Country : Japan Site: Single-center	Total: 41 patients who showed an inadequate response to biological DMARDs	IGU 25mg Qd (0-4 weeks), then increased to 25mg Bid based on the physician’s discretion.	24 weeks	remission can be achieved by IGU addon in RA patients responding partially to 24-week or longer administration of bDMARD
Zheng et al., 2018	Retrospective study	Country : China Site : Single-center	Total: 23 patients who showed an inadequate response to MTX–CsA–HCQ– prednisone	MTX:12.5mg Qw HCQ:0.1mg Bid CsA:50mg Bid Prednisone:7.5mg Qd IGU: 25mg Bid	24 weeks	After 24 weeks:the RA patients showed a significant improvement in mean DAS28 score from baseline. 18 (78%), 15 (65%), and 12 (50%) patients, respectively, met the ACR20, ACR50, and ACR70 response criteria.
Ebina et al., 2019	Retrospective study	Country: Japan Site: multicenter	Total: 31 patients who showed an inadequate response to TCZ	TCZ 162mg Q2w or 8 mg/kg Qm;IGU 25 mg Qd,then increased to 25mg Bid depending on physician’s decision.	24 weeks	Using the EULAR criteria, 64.5% achieved a moderate response, and 51.6% achieved ACR 20 at 24 weeks
Suto et al., 2019	Retrospective study	Country : Japan Site:multicenter	Total: 69 IGU group:28 MTX+IGU:28 bDMARDs+IGU:13	IGU group:IGU 25mg Qd (0-4 weeks), then increased to 25mg Bid based on the physician’s discretion. MTX+IGU group: MTX was 8.5 ± 3.4 mg/week bDMARDs+IGU group: IFX/ETN/ADA/TCZ/ABT/GLM(n=1/4/3/1/2/2)	36 months	The survival rate of IGU therapy at 3 years was 40.6%. The disease activity was significantly decreased in the IGU group and MTX plus IGU group compared with the baseline.

The authors apologize for this error and state that this does not change the scientific conclusions of the article in any way. The original article has been updated.
